# Development of Innovative Thermoplastic Foam Materials Using Two Additive Manufacturing Technologies for Application in Evaporative Cooling Systems

**DOI:** 10.3390/polym16223190

**Published:** 2024-11-16

**Authors:** Jesús Castillo-González, Francisco Comino, Roberta Caruana, Manfredo Guilizzoni, Paula Conrat, Manuel Ruiz de Adana, Francisco J. Navas-Martos

**Affiliations:** 1 Centro Tecnológico del Plástico Andaltec, Ampliación Polígono Cañada de la Fuente, Calle Vilches 34, 23600 Martos, Spain; jesus.castillo@andaltec.org; 2 Departamento de Mecánica, Escuela Politécnica Superior, Universidad de Córdoba, Campus de Rabanales, Antigua Carretera Nacional IV, km 396, 14071 Córdoba, Spain; francisco.comino@uco.es; 3 Department of Energy, Politecnico di Milano, Via Lambruschini 4, 20156 Milano, Italy; roberta.caruana@polimi.it (R.C.); manfredo.guilizzoni@polimi.it (M.G.); 4 Departamento de Química-Física y Termodinámica Aplicada, Escuela Politécnica Superior, Universidad de Córdoba, Campus de Rabanales, Antigua Carretera Nacional IV, km 396, 14071 Córdoba, Spain; z12coesp@uco.es (P.C.); manuel.ruiz@uco.es (M.R.d.A.)

**Keywords:** foam material, chemical blown agent, additive manufacturing, evaporative cooling, water absorption

## Abstract

Evaporative cooling systems have emerged as low-energy consumption alternatives to traditional vapor compression systems for building air conditioning. This study explored the feasibility of utilizing polymeric foamed materials produced through additive manufacturing as wetting materials in evaporative cooling systems. Specifically, two different commercial polylactic acid filaments, each containing a percentage of a chemical blowing agent, were studied. Experiments were designed to evaluate the influence of critical process parameters (line width, flow rate, speed, and layer height) on the performance of the resulting foamed materials in terms of evaporative cooling by conducting water absorption, capillarity, porosity, and wettability tests. Considering that high water absorption, capillarity, and porosity, coupled with an intermediate contact angle, are advantageous for evaporative cooling effectiveness, a low flow rate was found to be the most important parameter to improve these properties’ values. The results showed that the appropriate combination of polymer and process parameters allowed the production of foamed polymer-based materials processed by additive manufacturing technology with optimal performance.

## 1. Introduction and State of the Art

The demand for cooling in buildings has sharply intensified with the increase in the Earth’s surface temperature [1]. In addition, the energy demand for building refrigeration is expected to triple by 2050 [2]. Most heating, ventilation, and air conditioning (HVAC) equipment is based on conventional vapor compression cycles, which are characterized by high energy demand [3]. For this reason, evaporative cooling (EC) systems have been presented as an interesting low-energy consumption alternative to air condition buildings [4,5]. Some previous research works have incorporated HVAC technologies with EC to reduce the energy consumption of HVAC systems and thus enhance their efficiency, for example, (1) the combination of EC with and mechanical vapor compression systems [6]; (2) systems that synergistically combine thermal insulation, EC, and radiation cooling, reaching a temperature reduction up to 9.3 °C below ambient temperature with lower energy consumption compared to simple EC systems [7]; (3) HVAC systems based on EC, solar energy, and drying materials, with 75% of the energy consumed from renewable sources [8]; (4) systems that integrate a solar chimney, a wind tower, and EC, achieving a 70% reduction in operating costs compared to conventional systems [9]. To optimize the efficiency and cooling capacity of EC systems, some studies focused on enhancing the design, geometry, water distribution, and implementation of new porous materials [10]. Other authors proposed incorporating new internal structures, such as fins and corrugated sheets, to improve heat transfer, as well as using optimized spray systems to improve wettability [11].

**Figure 1:**
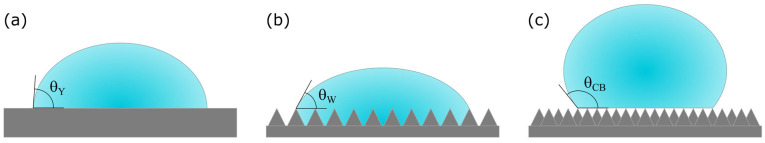
Schematic representation of a drop (**a**) on a flat smoot substrate, with the intrinsic Young’s contact angle; (**b**) on a rough substrate in the Wenzel wetting state, with the apparent Wenzel contact angle; (**c**) on a rough substrate in the Cassie–Baxter wetting state, with the apparent Cassie–Baxter contact angle.

In the literature, strategies for improving EC technology have been presented. As examples, the following can be cited: (1) using metal foam as a material [12], (2) combining EC with desiccant material technology [10], (3) improving water distribution in a porous material [10], (4) developing new porous materials [10], (5) optimizing the spray systems [11], and (6) exploring low-cost, long-life materials with good water retention capacity [11]. In terms of characterizing wetting materials, capillarity height, diffusivity, and evaporation rate are the three most important indicators for evaluating their performance in EC systems [12]. It is remarkable that the greater the rate and volume of the water absorbed, the greater the cooling generated [13]. Wettability is also crucial, as poor wettability results in low air cooling [11], as the uniform and complete wetting of the surface increases the mass transfer area and decreases thermal resistance [2]. Surface engineering may also play a role, as the interactions between liquids and solids on textured or rough surfaces differ from those where the solid substrate presents a smooth surface [14]. A drop in contact with a textured surface can adopt different configurations, usually named as the Wenzel and Cassie–Baxter wetting states (Figure 1) [15]. Some surfaces may also exhibit a transition between the Wenzel and Cassie–Baxter configurations due to an energy barrier from both states, which could occur suddenly or smoothly [16].

Wettability is generally characterized by using static contact angles (*θ* in Figure 1)—either the theoretical Young’s contact angle *θ_Y_* or the apparent contact angles *θ_W_* (Wenzel contact angle) and *θ_CB_* (Cassie–Baxter contact angle), as well as the dynamic contact angles for EC systems [17]. Some authors have developed specific methods for the characterization of materials in terms of EC capacity, utilizing wind tunnels designed specifically for this purpose [18,19]. These wind tunnels allowed control over key parameters such as air temperature, wind flow, relative humidity, and solar radiation in order to measure the evaporation rate and cumulative evaporation of the materials studied.

The water absorption rate driven by capillary forces is a useful metric for assessing a material’s water absorption capability [20]. Therefore, several researchers have studied the capillarity height to determine the EC capacity of materials [20,21,22]. The capillarity is ruled by micropore distribution and surface tension, while the evaporation rate is determined by the macropore structure [22]. As such, pore structure has been widely studied in terms of EC [12,20].

For the selection of the most suitable wetting material, high porosity, a large specific surface area, and small tortuosity should be the main properties to take into account [12]. Fibers have been widely proposed as wetting materials in the literature: cellulose, coconut, palash, palm, tossa jute, luffa, poplar wood, Kraft, eucalyptus, cellulose and PET composite, coolmax cloth, wood pulp paper, and felt paper [13,23]. In general, these fibers exhibit good permeability, allowing water to evaporate from a wet surface.

Regarding the methods of joining the wetting material of the wet channel and the material of the dry channel, heating and gluing technologies have generally been used, which present economic and environmental disadvantages [13]. An alternative method of manufacturing EC is additive manufacturing (AM) technology. Several studies have explored the use of polymer-based AM for the fabrication of heat exchangers using polymer AM technology [24,25,26]. However, in terms of EC system manufacturing, only the study carried out by Castillo et al. [4] was found in the literature.

The manufacturing of heat exchangers using AM techniques allows much greater design and manufacturing freedom than other conventional manufacturing techniques [27,28,29]. Furthermore, AM offers significant potential to combine multiple materials and adapt their properties to the requirements of different products [30]. Although the low thermal conductivity of materials used in AM (0.3–0.5 W/m K) could present a limitation [31], previous research showed that when thicknesses of less than 0.5 mm are used, the heat transfer resistance of materials becomes negligible [32]. The production of an EC system requires the production of porous structures; AM perfectly fulfils this requirement, providing precise control over pore geometry owing to its inherent design flexibility and the ability to modify processing parameters [29]. The techniques for generating porous structures through AM can be classified into different types [33,34]. Type 1 includes porosity from the beginning in the original 3D design of the part. Type 2 generates porosity through the addition of fibers or fillers. Type 1 and Type 2 can be considered prefoaming manufacturing techniques because foaming occurs before AM. Type 3 and Type 4 are characterized by the generation of the foamed structure taking place after and during AM, respectively. Additionally, Type 5 can also be considered, which consists of modifying the AM parameters, such as the distance between rods, layer height, or rod width, in order to increase the porosity [33]. Concerning Type 1, the following research works are notable examples: (a) the development of strategies with low computational cost for the generation of customized internal porous structures and specific external surfaces [35]; (b) the combination of X-ray microcomputed tomography, digital pore modeling, and AM technology to transform digital models into real parts, achieving resolutions of up to 7 μm [36].

Regarding Type 2, some studies stand out: (a) the development of an innovative prototype of an EC system manufactured using AM technology, generating a porous layer by dissolving a polymer matrix (polyvinyl alcohol), where a porous material was initially introduced [4]; (b) the AM of parts initially composed of a thermoplastic polymer composite with nickel powder as a filler, with these resulting parts being subsequently subjected to heat treatment to eliminate the polymer matrix to sinter the nickel particles and finally obtain a porous open cell structure [37]; (c) the manufacture of foamed materials using AM by adding glass micro-balloons to the AM filament [38]; (d) the manufacture of porous filter of organic molecules by 3D printing using a high-impact polystyrene filament obtained by adding active carbon to the polymer [39]; and (e) the use of AM and Lay-Felt filament to manufacture a porous membrane with a gasket-less design [40].

Considering Type 3, precast parts are first manufactured via AM without a cellular structure and then saturated with a gas at high temperature and pressure [41]. Some examples are remarkable: (a) the manufacturing of thermoplastic polyurethane foam with CO_2_ as a foaming agent [42]; (b) the manufacturing of porous structures based on polylactic acid (PLA) with two different levels of porosity, namely, a macroporous PLA structure (macropores of 100–800 μm manufactured by modifying AM parameters) and micropores (from 1 to 10 μm) generated with gas foaming techniques [43].

In relation to Type 4, foaming occurs during AM, using filaments containing chemical blowing agents (CBAs) or physical blowing agents (PBAs) [41]. Some related works are highlighted: (a) the manufacturing of PLA microfoams using CO_2_ as PBAs, achieving the greatest weight reduction at a CO_2_ content of 15 wt% [36]; (b) the manufacturing of a foamed composite using PBA composed of nanocarbon, Fe_3_O_4_, polyether-ether-ketone (PEEK), and polyetherimide (PEI) [44]; (c) the development of a new process to produce thermoplastic polymer foams with PBA solubilized during the AM, achieving better geometry resolution, greater flexibility, and lower process cost [45]; (d) the development of polyetherimide (PEI) filament with PBA [46]; (e) the use of cork to improve the properties of polyurethane (PU) foams manufactured using filament with PBA [47]; and (f) the manufacturing of porous parts by means of AM using two strategies in parallel: first, generating macroporosity by simply separating the printing beads from each other and, second, using filaments with CBA [33,34]. Notably, the gas concentration in the filament and the nozzle temperature during the printing process are critical parameters controlling the foam density, bubble density, and crystallinity [48]. In addition, when it comes to PBA, it is very important to control the time from gas introduction to printing (desorption time) due to the gas being gradually lost [44]. Comparing CBA and PBA filaments, the main problem with those containing PBA is maintaining the gas in the filament until it is processed. CBA does not have this problem; however, it is not as efficient for pore generation as PBA [41].

Regarding Type 5, some remarkable studies are (a) the manufacture of porous sound-absorbing parts using the “fiber bridging method”, which involves the continuous extrusion of filament between two points without underlying support, or the “extrusion and pull method”, which requires the extrusion of a small amount of heated filament before the printing nozzle is rapidly withdrawn to generate thin fibers [49]; (b) the manufacture of porous structures by AM, mainly by modifying the distance between rods [50]; (c) the study of the effects of modifying the layer height on the mechanical and water absorption properties of parts made of PLA loaded with vegetable fiber [51]; (d) analyses of the influence of modifying the AM parameter “print width” on the mechanical properties and microstructure of wood biocomposites [52]; (e) the manufacture of porous structures with two materials (acrylonitrile butadiene styrene and PLA) by means of AM, controlling the porosity with different process parameters and achieving pores between 200 μm and 300 μm [53]; and (f) the analysis of the influence of the infill rate on the porosity and biocompatibility of bone tissue scaffolds [54].

Furthermore, other researchers such as Choi et al., [33], Marascio et al. [34], and Zhou et al. [43] applied Type 5 alongside other methods. In summary, the main 3D-printing parameters that influence the mechanical properties and porosity are infill percentage, filament thickness, layer height, distance between rods, raster angle, and external geometry [53]. Water absorption increases while swelling decreases with increasing layer height, although this reduces the mechanical properties [51]. Increasing the distance between rods improves porosity and water absorption but also causes more swelling and reduces cohesion, leading to a reduction in mechanical properties [52].

Therefore, a potential strategy to improve HVAC systems based on EC technology is to develop new solutions in terms of wetting materials. The effect of water absorption by capillarity is generated with particle sizes between one and a few hundred microns [55]; hence, the strategy of manufacturing porous structures using mixing AM Type 4 and Type 5 [33,34] techniques perfectly meets the requirements of HVAC systems in terms of wetting materials. Thus, the main objective of the present work was to explore the feasibility of developing foam materials using fused deposition modeling (FDM) technology for EC applications. The Type 4 technique, i.e., the development of new foam materials using CBA and PLA, and the Type 5 technique, i.e., the development of new foam materials by means of modifying process parameters, were used. No previous research by other authors on the use of polymer-based foamed materials obtained by AM techniques specifically for EC applications was found in the literature.

## 2. Material and Methods

### 2.1. Selection of Materials and Manufacturing of Samples

Two commercial polymeric materials based on PLA with CBA were selected to develop in situ foam materials through AM: (a) white Filaticum Foam (DW) and (b) black BigRep PLA (DB), supplied by Filamania KFT (Miskolc, Hungary) and Bigrep GMBH (Berlin, Germany), respectively. Both materials were provided in filament format with a diameter of 2.8 mm to allow suitable processing using AM technology with a UltiMaker 5S 3D printer (UltiMaker, Utrecht, The Netherlands). The AM method used, FDM, involved creating a 3D design of the part to be manufactured, which was then processed using specialized software (UltiMaker Cura 5.8, Utrecht, The Netherlands) prior to the printing process. Finally, the 3D object was obtained by extruding the thermoplastic material layer-by-layer. The final structure of the foam materials as well as its quality and surface morphology could be modified by adjusting the print settings.

In this work, the two selected foam materials were characterized under different manufacturing conditions. A Taguchi L9 design of experiment (DOE) was carried out considering five critical parameters of the manufacturing process: (a) line width (LW), i.e., the separation between the paths within each layer, ranging from 0.4 mm to 0.8 mm; (b) flow rate (F), namely, the volume percentage of extruded material with respect to the maximum that could have been extruded, varying from 50% to 100%; (c) speed (S), i.e., printer head speed, ranging from 40 mm/s to 80 mm/s; and (d) layer height (LH), namely, separation between layers, ranging from 0.2 mm to 0.4 mm. The selection of these four parameters was established after carrying out some preliminary trials, from which it was elucidated that LW and F showed a strong correlation with porosity in the AM processes, while S and LH were critical parameters ensuring proper manufacturability. The printing temperature could also be considered essential; however, it was not included as a variable because the suppliers recommended using a constant temperature of 250 °C. A total of 9 samples were obtained from each foam material by modifying the process parameters: samples from DW1 to DW9 were based on DW, and samples from DB1 to DB9 were based on DB. Table 1 summarizes the values used for each parameter in every case. The considered parameters were the same for the DW and DB samples; therefore, the nomenclature used to define each case was D1 to D9. D_ref_ was manufactured for both kind of samples, DW and DB, with the aim of studying a set of parameters that generated a poreless sample.

### 2.2. Characterization of Foam Materials

Several experimental tests were carried out for each sample of material to produce the greatest EC effect. The characterization of the foamed material specimens was based on (a) capillary rise tests, to obtain the ability of these materials to transport and retain water in their porous structure; (b) water absorption tests by immersion, to know the amount of water that the materials can retain; (c) surface analysis by using scanning electron microscopy (SEM) and two different types of porosity tests, the first based on nitrogen adsorption–desorption isotherms, allowing the determination of the surface area and the amount and size of pores, and the second based on water vapor adsorption–desorption isotherms, allowing the determination of the surface morphology; and (d) wettability tests to know how water interacts with the surface of materials. All the specimens shown in Table 1 underwent analyses for capillarity and water absorption. A statistical analysis was conducted to comprehensively interpret the results. Based on the results of this analysis, a selection of 3 specimens based on DW and 3 based on DB were analyzed in terms of porosity and wettability. This selection aimed to reduce the number of tests to be performed.

#### 2.2.1. Capillarity

**Figure 2:**
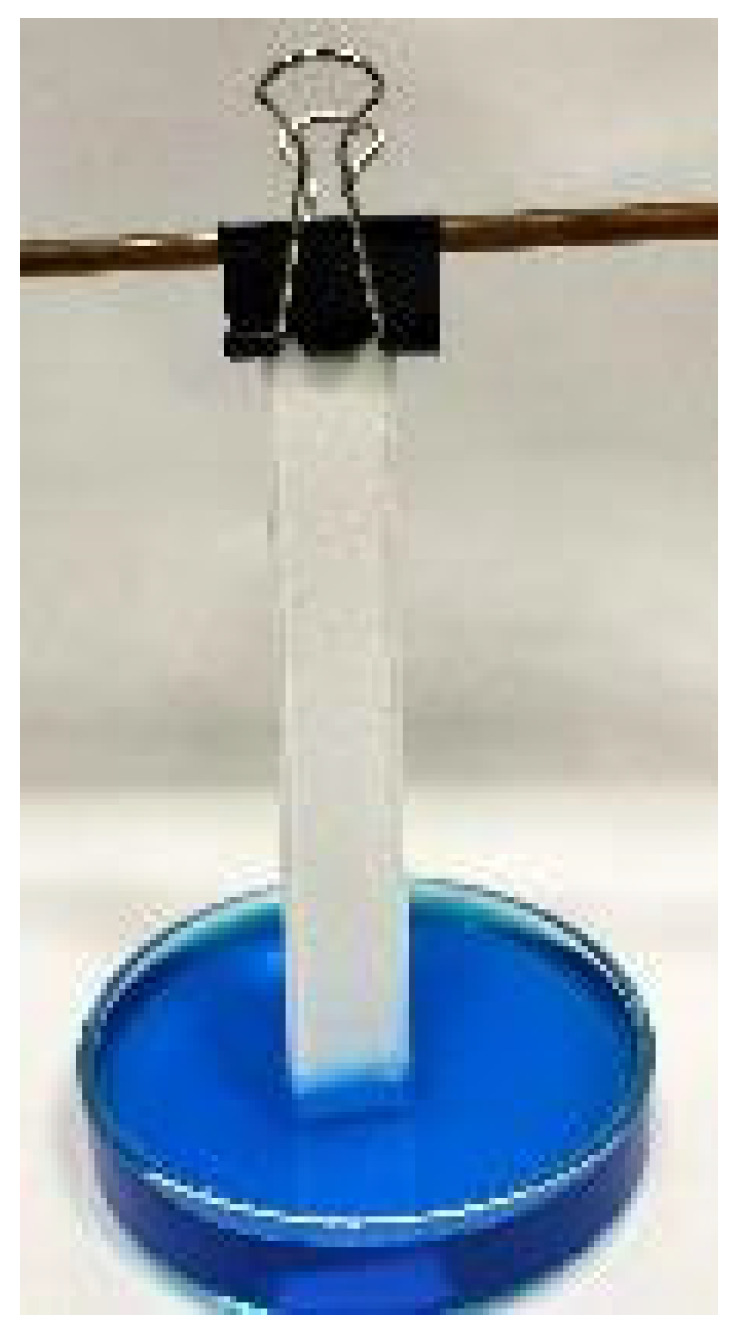
Setup for capillary rise tests.

The capillary rise tests were carried out using 80 × 15 × 0.8 mm specimens, as shown in Figure 2. For each specimen, the capillary rise height (CH) of the water in the material was measured, which is related to the capacity to retain water. To clearly visualize the CH reached by the fluid in each sample, a blue dye was added to the water, not significantly influencing the density or surface tension of the fluid. The tests followed the methodology described in similar studies [2,12]. The specimens were placed vertically in a water bath. The tests ended when it was verified that water stopped rising through the samples (approximately 2 h after starting the tests). For this verification, periodic measurements were taken every 5 min using a caliper with a measurement accuracy of 0.1 mm.

Further analyses of the capillary rise data were performed using the classic capillary rise models along smooth flat plates (Equations (1) and (2)) and in capillary ducts (Equations (3) and (4)) [56]:

(1) $$CH=L_{c}\sqrt{2\left(1-\sin\theta_{Y}\right)}\;with\;L_{c}=\sqrt{\frac{\sigma }{\rho g}}$$

(2) $$\theta_{Y}=\;\sin^{-1}\left[1-{\frac{\left(\frac{CH}{L_{c}}\right)}{2}}^{2}\right]$$

(3) $$CH=\frac{2\sigma \cos\theta_{Y}}{\rho gR_{pore}}$$

(4) $$R_{pore}=\frac{2\sigma \cos\theta_{Y}}{\rho gCH}$$

where ρ is the fluid density, σ is the fluid–gas surface energy, g is gravity, $R_{pore}$ is the equivalent pore radius, and $L_{c}$ is the capillary length.

#### 2.2.2. Water Absorption

**Figure 3:**
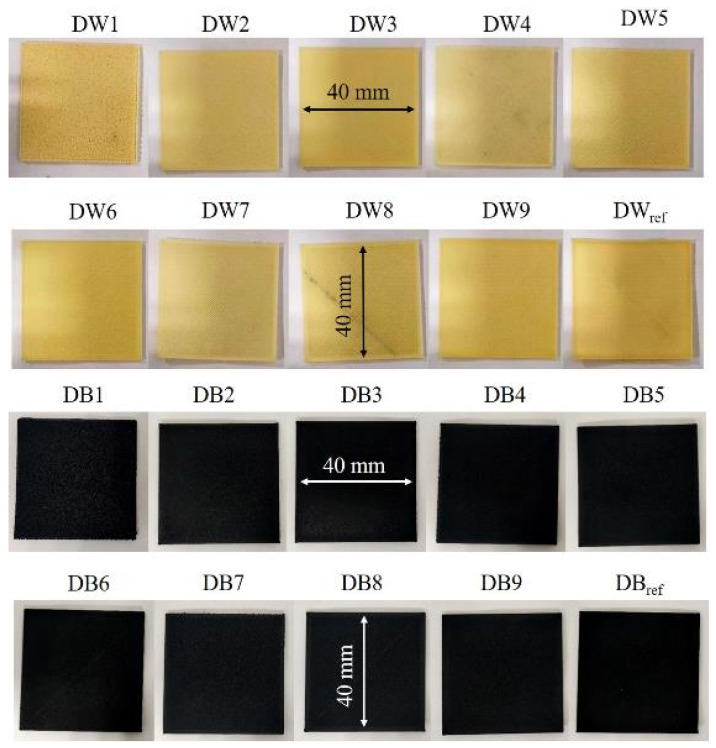
Samples of DW (**top**) and DB (**bottom**) manufactured for the water absorption tests.

The water absorption tests were performed according to the methodology described in method 1 of the ISO 62 standard [57] and using the kind of specimen shown in Figure 3, with a size of 40×40×0.8 mm. The samples were dried for 24 h at 50 °C before being weighed for the first time and then immersed in distilled water at 23 °C. A precision scale (U.S. Solid, UUEE, Cleveland, OH, USA) with a measurement accuracy of 0.1 mg was used for weighing. The ISO 62 standard establishes the weighing of the samples during the test after defined periods of time (24, 48, 96, 192 h, etc.) to obtain the amount of water absorbed by each material. However, since the water saturation of the DW and DB specimens occurred quite rapidly, the time periods for weighing were adapted in this work to 1, 5, 10, 20, 30, 40, 50, 100, 200, 300, 400, and 500 min. The results obtained from these tests were (a) the total water mass absorbed (I_m_) and (b) the water absorption rate (I_r_).

#### 2.2.3. Surface Analysis

The porosity of the materials was obtained from nitrogen adsorption–desorption isotherm tests. These tests allowed studying the surface of the materials in terms of (a) surface area (SA); (b) total pore volume of adsorption (PVA), considering pores with sizes of less than 403.12 Å; (c) total pore volume of desorption (PVD), taking into account pores with sizes of less than 25.88 A; (d) cumulative pore volume (CPV); (e) average pore width of adsorption (APWA) and desorption (APWD); (f) quantity of water adsorbed (QWA); and (g) differential pore volume (DPV), considering the amount of pores as a function of pore size.

**Figure 4:**
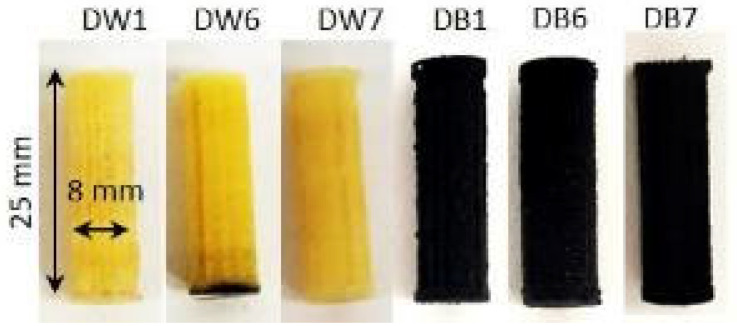
Samples of DW (**left**) and DB (**right**) manufactured for the porosity tests.

Cylinder-shaped specimens, 2.5 cm in height and 0.8 cm in diameter at the base, were fabricated to carry out these tests (Figure 4). A Micrometrics 3Flex Surface Characterization System (Micromeritics, Lincoln, UK) with a measurement accuracy of 0.1 bar was used to obtain the results. The tests were carried out by modifying the relative pressure of the gas (P/Po, where P is the test pressure, and Po is the saturation vapor pressure of the gas) at a constant temperature of 77 K. This system allowed the characterization of the porosity of the specimens in terms of micropores and mesopores. However, to observe the macropores, digital images were taken using a camera (EOS 2000D APS-C, Canon, Tokyo, Japan).

Water vapor adsorption–desorption isotherm tests were also carried out with the objective of identifying the material with the highest water vapor adsorption capacity and estimating the surface morphology of the pores generated. The samples and test system used were the same as for the nitrogen isotherms, under specific thermal conditions at 298 K.

**Figure 5:**
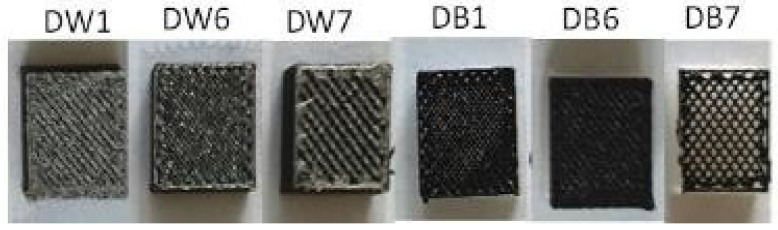
Samples of DW (**left**) and DB (**right**) manufactured for the wettability and SEM tests.

The microstructure of the different types of specimens was analyzed by SEM using a JSM-7800F (Jeol, Tokyo, Japan) to observe their morphologies, pore sizes, and general microstructural characteristics. For this purpose, square geometry samples with a 10 mm side and a 4 mm thickness (Figure 5), were prepared for six different materials, which were finally coated with a thin layer of gold to render them conductive and suitable for SEM analysis.

#### 2.2.4. Wettability

Six rectangular-shaped samples (10×10×5 mm) of each material (DW1, DW6, DW7, DB1, DB6, DB7) were manufactured to carry out the wettability tests (Figure 5). The testing methodology was the same as that followed in previous works for the evaluation of the contact angles [17,58]. The static contact angle was analyzed by depositing sessile drops on the surfaces, taking side-view macrophotographs of these drops, and measuring the contact angle using the axisymmetric drop shape analysis (ADSA) technique. FDM technology generates micro-grooves oriented along the printing direction of each layer, constituting the upper layer of each manufactured specimen, an asymmetric substrate on which the drops are partially elongated according to the Gibb’s “pinning on sharp edges” effect. Previous tests showed that ADSA can still provide a suitable approximation of the contact angle values [59], but, to have more detailed information on the latter, the analysis was performed by taking the pictures with the camera lens positioned both parallel and perpendicular to the printing direction of the upper layer of the specimen. The drop contour was extracted using image-processing techniques, and the static contact angle was evaluated by fitting the Laplace–Young equation to the drop boundary. The resulting contact angle value obtained for each specimen was used as a comparative measure of the wettability of the samples. Further details about the techniques used and their measurement accuracy can be found in [60,61].

Finally, the Cassie–Baxter model was used (according to Equations (5) and (6)) to estimate the wetted fraction for each of the 6 surfaces analyzed, where f is the fraction of the surface wetted by the liquid. The Cassie–Baxter model can be considered the best compromise between simplicity of use and accuracy of the results, being reliable for polymers with a morphology that is partially similar to those of the surfaces analyzed in this work [62,63].

(5) $$\cos\theta_{CB}=f\cos\theta_{Y}-\left(1-f\right)$$

(6) $$f=\frac{\cos\theta_{CB}+1}{\cos\theta_{Y}+1}$$

## 3. Results and Discussion

### 3.1. Analysis of Water Absorption by Capillarity and Immersion

**Figure 6:**
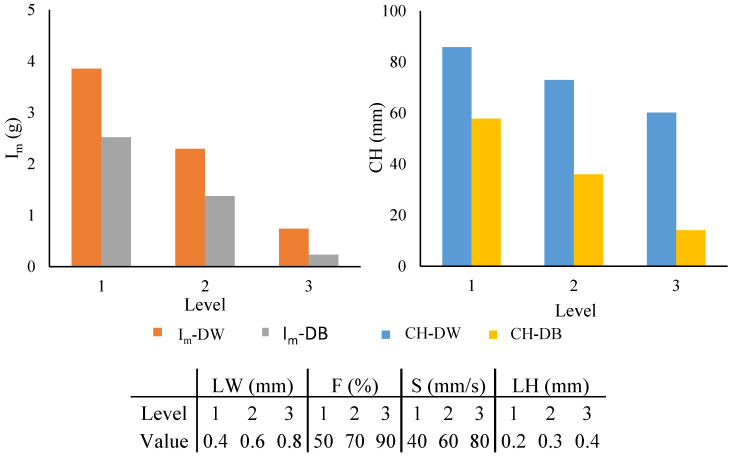
Influence chart obtained from the statistical analysis of the DOE results.

The influence of each process parameter (LW, F, S, and LH) on the water absorption by the capillarity (CH) and immersion (I_m_) of both materials (DW and DB) obtained from the results of the statistical analysis is shown in Figure 6. Level 1 represents the minimum value of all parameters studied in the DOE (Table 1), level 2 is the medium value, and level 3 is the maximum value. It was observed that a reduction in the level, and thus in the value of all parameters, generated improvements in the CH and I_m_. This improvement was the greatest in DW, where increases of 3 g in the I_m_ and 60 mm in the CH was observed from level 3 to level 1. However, a less significant improvement was observed for DW, being only 1.2 g in I_m_ and 25 mm in CH. Concerning I_m_-DB and CH-DB, a similar improvement was observed after modifying the parameter: specifically, an increase of 2.25 g in I_m_ and of 45 mm in CH. Concerning the evaluation of the fit of the statistical model with the experimental data, the R^2^ parameter was employed. A better correlation was observed for CH-DW and Im-DW than for CH-DB and Im-DB; more specifically, R^2^ values of 0.955, 0.938 versus 0.681, 0.861 were obtained, respectively.

It was also remarkable that not all input parameters had the same influence on the final results. To determine this representability, the statistical *p*-value was studied (Table 2): the greater the input parameter influence, the lower the *p*-value, with 0.05 being the maximum *p*-value that indicated that there was an influence. The *p*-value results showed that F was the most influential parameter in all cases studied. In contrast, the parameter with the least influence was S, which was not representative in any output parameter. LW was the second most influential parameter, showing a notable effect on CH-DW and I_m_-DW but not on any of the other. Finally, LH was the second least influential input parameter and presented a *p*-value greater than 0.05 only in CH-DW.

**Figure 7:**
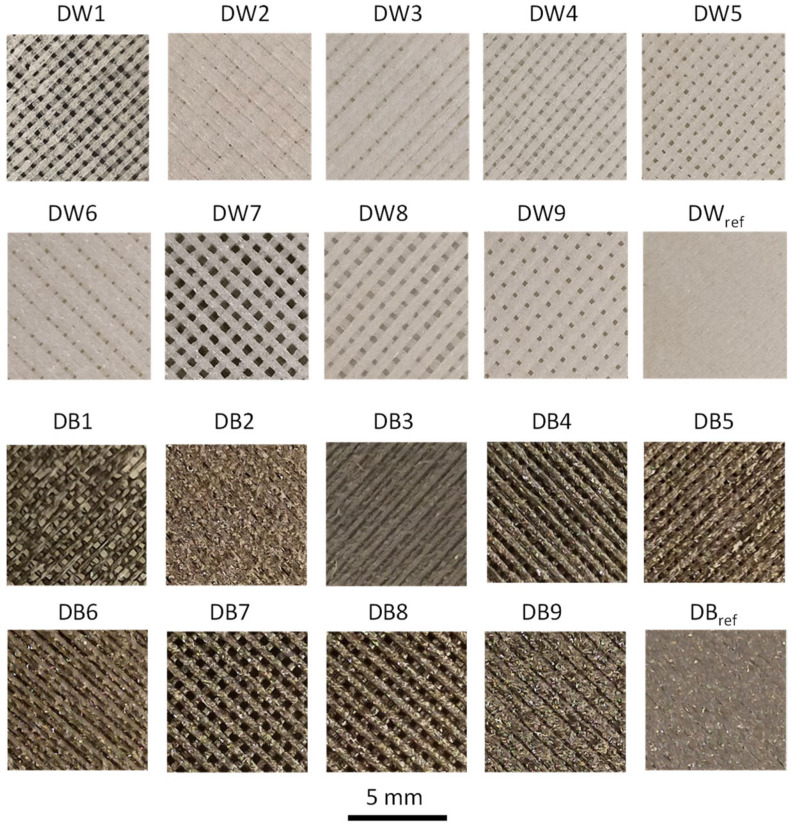
Images showing the macropores of all samples made with the DB and DW materials.

The surface macropore structure of each DW and DB sample is shown in Figure 7*,* showing the same trend for both materials. D1, D4, D7, and D8 were the specimens with the highest microporosity, which corresponded to manufacturing conditions with minimum values of F (50%), except for D8, where F was 70% and LW was 0.8 mm. The samples with lower microporosity (D2, D3, D6, and D9) had a higher F (90%), except for D2, where F was 70% and LW was 0.4 mm. Finally, although the trends in both materials were very similar, a slight difference was observed in terms of macropores: DW presented macropores in all samples except DW_ref_; however, DB did not present macropores in samples DB2, DB3, DB6, DB9, or DB_ref_.

**Figure 8:**
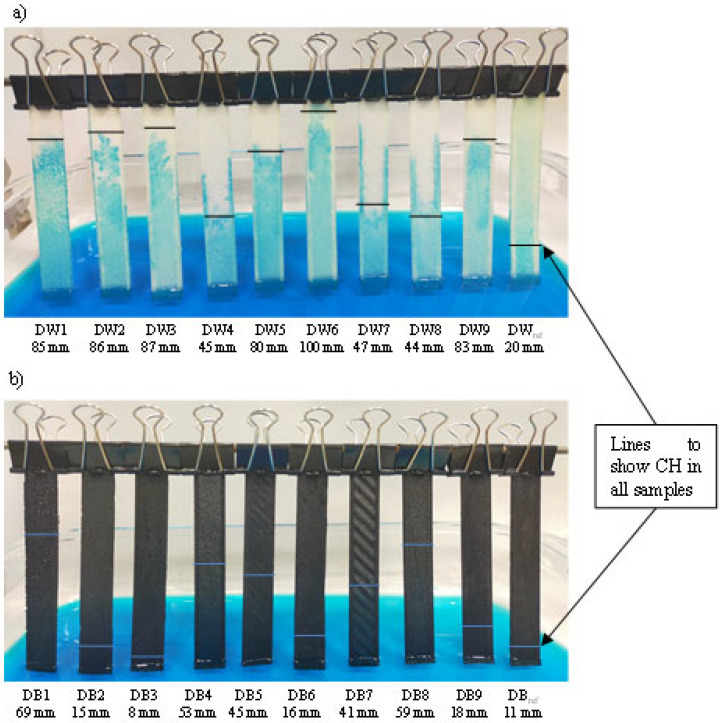
Capillarity tests of (**a**) DW and (**b**) DB samples.

The capillary rise height (CH) values achieved by each DW and DB sample are shown in Figure 8. In the case of the DW samples (Figure 8a), the water height was lower for DW4, DW7, and DW8, which presented larger macropores, according to Figure 7. This was mainly due to the fact that their macropores volumes were larger, so the gravitational force on the mass of the hosted water was greater than the force exerted by the capillary effect. Regarding the CH values for DB (Figure 8b), the samples DB1, DB4, DB5, DB7, and DB8 obtained results significantly higher than the rest due to the differences in pore sizes, which is the same that occurred with DW. Concerning the CH values obtained for DB_,_ they was much lower than those of DW. Finally, it was remarkable that the DB specimens that did not show any macroporosity (Figure 7) were the same that had the minimum CH values (DB2, DB3, DB6, DB9, and DB_ref_).

The capillary rise data obtained from the classic capillary rise models (Equations (1) and (2)) are presented in Table 3. It was found that even for a contact angle of 0°, the observed capillary rises could not have been reached on a smooth flat plate; therefore, a contribution either of surface grooves or pores was assumed. Considering Jurin’s law (Equation (3)) and making it explicit in the pore radius (Equation (4)), an estimation of the equivalent pore radius needed to reach the observed capillary rises was obtained.

In fact, in all the tests, the used fluid was water in a surrounding air atmosphere, both the DB and DW samples were based on PLA, and the temperature conditions were the same. Thus, it could be assumed that the surface energies and the intrinsic, chemical, contact angle $\theta_{Y}$ were the same for all cases. No wettability tests on flat, smooth samples were performed (because 3D-printed samples are never perfectly smooth), but as PLA is a commonly used material, its contact angle values have been reported in previous works, covering a relatively wide range, from 60° to 85°, due to the strong dependence on the roughness [64,65]. Given that the main aim of this work was to rank the specimens, a value equal to 60° was assumed as a reference for flat, smooth samples.

As it can be seen in Table 3, the specimens could be sharply clustered into two groups, the first with a pore radius under 200 microns (which was consistent with their visual aspect and the characteristics of the manufacturing technique, and the second with a pore radius from 370 to 900 microns. The samples in the second group were those visually identified as not showing macroporosity. For them, the rise being due to surface grooves, rather than pores, was most likely.

**Figure 9:**
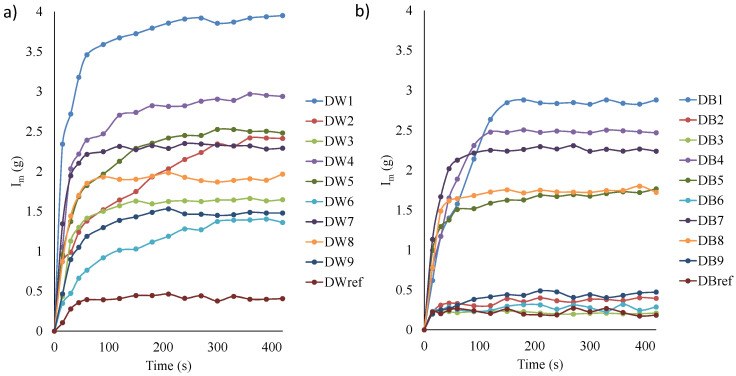
Water absorption test of (**a**) DW and (**b**) DB samples.

The amount of total water absorbed (I_m_) for each DW and DB sample is shown in Figure 9. The analysis revealed that the water absorption process could be divided into three phases. In phase 1, there was a high rate of water absorption. Here, water intake was influenced by both capillary and viscous forces. At this point, water had not yet permeated the innermost layer of the specimen. Phase 2 involved a transitional process, characterized by the continuous filling of the small cavities in the sample until it attained capillary saturation. In phase 3, water saturation was achieved; in the case of EC, it was the phase where the greatest mass transfer occurred between the air and the water retained by the material. Therefore, the objective was to achieve the highest I_m_ value in phase 3 in the shortest possible time. These three stages were also observed by Wang et al. [20] and Zang et al. [19].

The trends were similar for both materials; however, significant differences in values were observed: in general, DW absorbed around 30% more water than DB. Focusing on DW (Figure 9a), DW1 was the sample that absorbed the most water and DW_ref_ the least (material without pores). It was remarkable that, in most cases, phase 1 of I_m_ ended after the first 50 s. However, specimens DW2 and DW5 required 300 and 150 s to reach the end of phase 1, respectively. According to these results, the best samples were DW1 and DW7, which had the highest I_m_ values in a short period. Additionally, Figure 9a shows I_r_, which represents the rate of water absorbed (g/s). Therefore, by analyzing this figure, it can be seen that during phase 1 (first 50 s), the values of I_r_ were higher, given its high slope. I_r_ was higher for specimens DW1, DW4, and DW7, which corresponded to the manufacturing conditions with the minimum values of F (50%), leading to specimens with greater porosity, according to the images in Figure 7. Of these samples, DW1 had the lowest LW and presented a much higher slope than the others.

The results obtained from the configurations based on DB (Figure 9b) presented a significant gap between the results of the first group of samples, composed of DB1, DB4, DB5, DB7, and DB8, and a second group composed of the rest of the samples. Figure 7 clearly shows that the first group had high porosity, while the second did not present any representative porosity. Those configurations that did not present porosity barely absorbed water, and the height reached by the water in the capillary rise test was essentially negligible (Figure 8). In terms of I_m_ (Figure 9b), configurations DB1, DB4, and DB7 stood out significantly, ordered from highest to lowest. These configurations corresponded to specimens that had a larger macropore size (Figure 7). Moreover, it was remarkable that phase 1 finished in the first 50 s in all samples, except for DB1, which required approximately 110 s.

Focusing exclusively on the parameters studied (CH and I_m_) and on those samples that presented greater porosity, D1 was the one that had the best and most promising results, both in the case of samples based on DW and those based on DB. However, when comparing DB and DW, it was remarkable that DB did not produce the same results as DW_,_ either in the D1 configuration or in the other configurations.

### 3.2. Surface Analysis of the Foam Materials

A surface analysis of the foam materials was carried out for three DW specimens and three DB specimens, as previously indicated. The selected samples were D1, D6, and D7, which had the highest, lowest, and average values of absorption capacity by capillarity and immersion, respectively.

**Figure 10:**
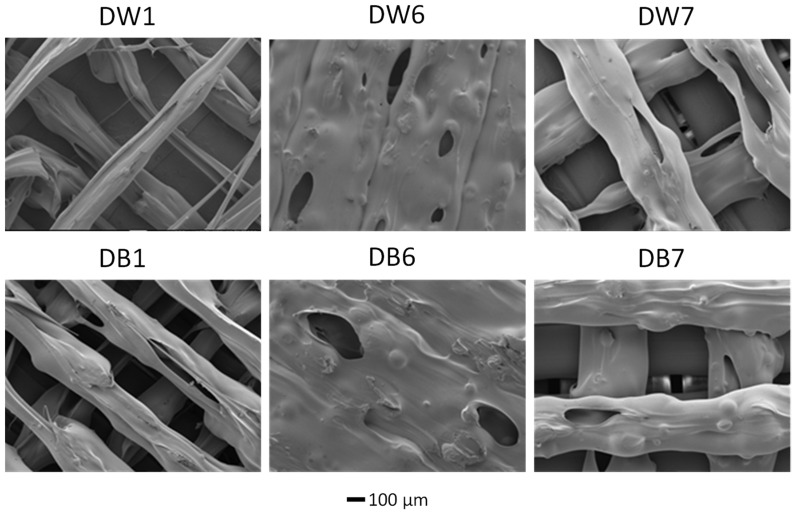
SEM images of samples DW1, DW6, DW7, DB1, DB6, and DB7.

The microstructures of the samples were noticeably affected by the changes in the manufacturing parameters, as shown in the SEM images in Figure 10. DW1 and DB1 exhibited a fibrous morphology with elongated pores and significantly narrower size in comparison to the other samples. This result was attributed to the smallest LW value (0.4 mm), which matched the nozzle diameter, along with an F of 50%, which was used during the fabrication process. The internal microporosity of the filaments was induced by the CBA, whereas the macroporosity resulted from the specific manufacturing settings. In contrast, for DW6 and DB6, solid regions with fused filaments formed, with no visible separation between the threads, mainly due to the increased F of 90%, the highest value used in this study. The results for DW7 and DB7, corresponding to the samples with the widest LW (0.8 mm), were well defined and uniform but presented a smaller pore size than DW1 and DB1.

**Figure 11:**
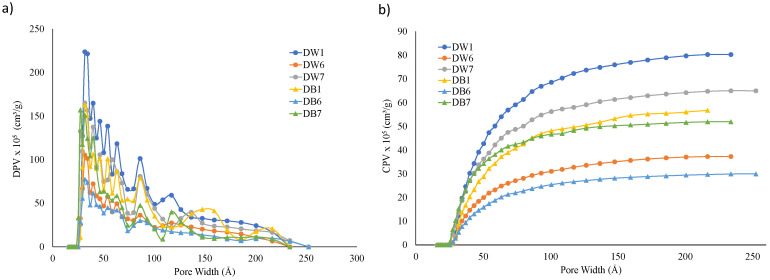
(**a**) DPV and (**b**) CPV vs. pore width.

Nitrogen adsorption isotherms tests were performed for surface analysis. The CPV and DPV f results or each specimen with respect the width of the pores generated are shown in Figure 11. It can be observed that these materials exhibited mesoporosity, mainly in the 20–200 Å range, as shown in Figure 11a. Pore width values of around 30 Å predominated in all samples, indicating that the majority of the pores had that dimension. However, the shape of the curves indicated that many pores with a width in the range between 30 Å and 100 Å were also generated, so the porosity of the samples was heterogeneous.

Regarding cumulative pore volume (CPV), the DW samples presented higher values than the DB samples, as shown in Figure 11b. This trend was similar to that obtained in the macropore structure shown in Figure 10. The D1 configuration presented higher CPV and DPV values than D7 and D6.

The isotherm tests also allowed us to obtain the values of SA, PVA, PVD, APWA, and APWD for each sample, which are summarized in Table 4. It can be observed that those configurations with higher SA values exhibited higher PVA and PVD values. That is, D1 presented the highest values of SA, PVA, and PVD, followed by D7 and D6 (see Table 4). These three samples had the lowest F value (50%). These results are in agreement with the results of the capillarity and water absorption tests by immersion, where the D1 sample presented greater water absorption than the D6 and D7 samples (see Figure 8 and Figure 9).

Comparing the foam materials, DW exhibited higher SA values than DB (21% more), resulting in higher PVA (32.6% more) and PVD values (48.5% more). These trends were in line with the results of CPV and DPV, as shown in Figure 11. The APWA and APWD values showed minimal variation across the different configurations, as shown in Table 4. Therefore, the manufacturing method generated different surface area values but similar average pore width values.

Considering those samples based on DW, DW1 presented the highest values of SA, PVA, and PVD, followed by DW7 and DW6, which was the same trend observed for DB_,_ as shown in Table 4. These results aligned with the output of water absorption and capillarity, that is, the higher the porosity, the higher the water absorption and capillarity effect.

**Figure 12:**
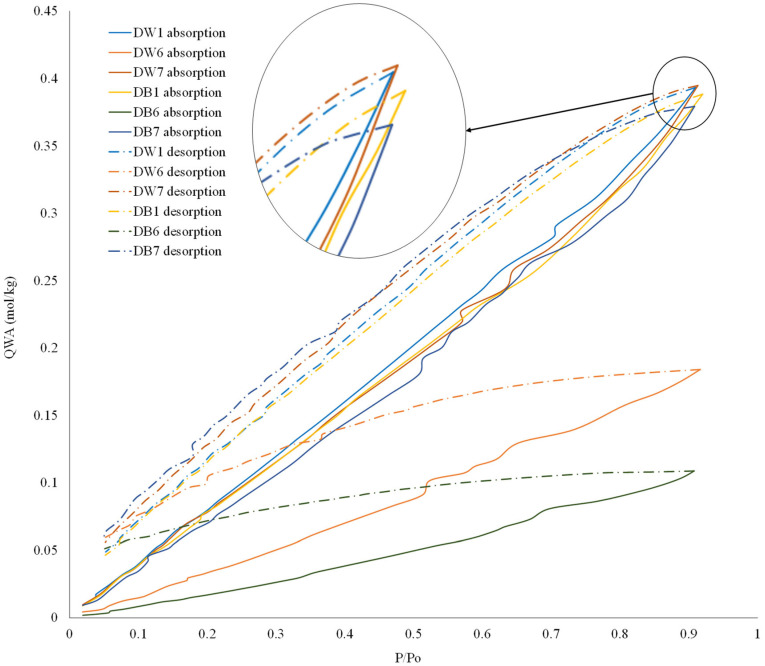
Results of water vapor adsorption isotherm tests for both foam materials.

The water vapor adsorption–desorption isotherms were obtained for each foam material to estimate the surface morphology. The results for the six materials are shown in Figure 12. In this figure, it can be seen that the isotherm trends were typical of meso- and macroporous materials. It was also significant that, as in previous analyses, the D6 sample presented lower absorption capacity than the other samples. However, in this case, the differences in the adsorption capacity between samples D1 and D7 were almost negligible, as well as the differences between DW and DB, as shown by the maximum values of P/Po at 0.9.

The morphology of the pores of these materials was estimated by analyzing the separation between the adsorption and desorption curves, given that the composition of all materials was the same (PLA). A larger difference between adsorption–desorption curves indicated that water vapor molecules were not as easily expelled during the desorption process. That is, when the difference between the adsorption–desorption curves was high, the pores of the material were closed or experienced a bottleneck; when this difference was low, the pores were open. In this study, DW1 and DB1 presented the closest adsorption–desorption curves, while DW6 and DB6 were the furthest apart, which justified the water absorption and capillarity results, where the DW1 and DB1 configurations presented much better results than DW6 and DB6. Moreover, it is notable that DB7 and DB1 presented a similar water vapor adsorption isotherm, in concordance with the results previously shown in Table 4.

### 3.3. Wettability

**Figure 13:**
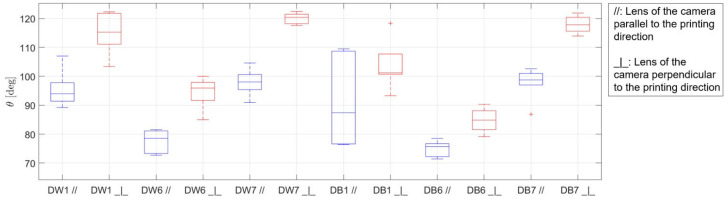
Boxplot showing the wettability results of all the samples studied: in the direction parallel to printing (//) in blue and perpendicular to printing (_┴_) in red.

The results in terms of contact angle obtained from the wettability tests are presented in the form of boxplots in Figure 13. The boxes show the first and third quartiles, with the central line being the median. The outer whiskers represent the maximum and minimum values, excluding outliers, which are represented by red crosses. Box plots are drawn in blue for results from the specimens whose grooves were oriented parallel to the camera lens and in red for the specimens whose grooves were oriented perpendicular to the camera lens.

**Figure 14:**
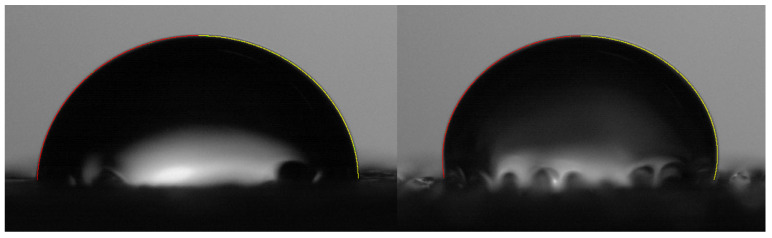
Images of the resulting drop contours (left contour in red and right contour in yellow), taken in the direction parallel (**left** picture) and perpendicular (**right** picture) to printing.

In general, it was first confirmed that the micro-grooves acted as barriers, with a “pinning on sharp edges” effect that made the contact angle measured in the orientation parallel to the printing path always lower than that in the perpendicular orientation. The evidence of what was described above was also supported by the photographs of the drops, as shown in Figure 14. This difference in contact angles due to the different orientations was already reported in the literature [65]. Therefore, when using FDM for EC system manufacturing, it would be advantageous to orient the grooves according to the direction of the water fall. In fact, the most suitable configuration in terms of wettability is probably an intermediate one because a surface that is too wettable can reduce the effectiveness of evaporation [66,67]; however, a surface that is too hydrophobic can prevent the formation of a suitable liquid film (favoring rivulet flow), thus reducing the surface available for evaporation and therefore the effectiveness [68]. Consequently, if the surfaces can be manufactured with directional roughness, this aspect can be taken into account during their design, namely, aiming to obtain the desired intermediate wettability. Moving to the comparison between the specimens, the D1 configuration presented intermediate values of contact angle; therefore, D1 presented the best set of parameters for improving the EC, in concordance with results of CH, I_m_, and porosity.

Concerning the literature, a reference value of 60° for the intrinsic static contact angle of PLA was found [64,65]. However, this was not the behavior observed in these tests, since all samples showed contact angles greater than 60°; this discrepancy could be explained by the rough surface of the samples.

Commonly, the intrinsic wetting behavior is enhanced when a Wenzel wetting state is reached; thus, a hydrophilic surface becomes more wettable, while a hydrophobic one becomes less wettable. However, in this case, the opposite behavior was observed. Therefore, Cassie–Baxter behavior was considered for all the samples, which was expected given their particular surface morphology (Figure 10). Assuming 60° as the intrinsic angle and taking into account the measurements of the apparent contact angle, the Cassie–Baxter model was used to estimate the wetted fraction for each of the six surfaces analyzed. The results are reported in Table 5, where, for each surface, the average value between the parallel and perpendicular directions was calculated. The results were in very good agreement with the visual observation, and, as can be observed from Figure 7 and Figure 10, the samples that visually showed larger pores were those with lower calculated wet fractions, consistent with a Cassie–Baxter wetting state.

The previous values were also consistent with the fact that the capillary rise tests effectively showed increases and not decreases. Thus, the in-pore contact angle was lower than 90° for all samples.

## 4. Conclusions

The present study examined the suitability of using foam polymer materials, produced through additive manufacturing technology, as wetting materials in evaporative cooling systems. Specifically, two different commercial polylactic acid filaments with chemical blowing agents were studied: DW and DB foam materials. A series of experiments were conducted to assess the impact of critical process parameters (line width, flow rate, speed, and layer height) on the performance of evaporative cooling systems through different tests: water absorption, capillarity height, porosity, and wettability.

The key findings of this study were as follows:

- Regarding water absorption and capillarity height, the flow rate was identified as the most influential process parameter, followed by line width, while the effects of the layer height and the speed were negligible. The optimal process parameters for maximizing the capillarity height and water absorption for both foam materials, DW and DB, were achieved with the following specific set of parameters: 50% flow, 0.4 mm line width, 40 mm/s speed, and 0.2 mm layer height.
- In terms of accumulated micropores and mesopores, the trends observed across different configurations were similar to those in the capillarity and water absorption tests. Samples with the D1 configuration presented greater surface area (21% more) and porosity (40% more), which resulted in a taller capillarity height and increased water absorption. These outcomes aligned with the results from the water adsorption and capillarity tests; that is, the greater the porosity, the increased the water absorption and capillarity effect.
- The results of the wettability tests were consistent with those of the capillary rise and macropore analyses. They demonstrated that the surface wetting behavior of the samples, characterized by roughness and micro-grooves due to the manufacturing technique, could be attributed to a Cassie–Baxter wetting state. Considering an intermediate contact angle as being advantageous for improving the evaporative cooling effect, the D1 configuration presented the best results, which aligned with the findings regarding capillarity height, water absorption, and porosity. Additionally, the wettability tests in both the along-grooves and cross-grooves directions demonstrated that the contact angle in the direction parallel to the printing orientation was always lower than that in the perpendicular orientation, as expected. Thus, the orientation of the material within evaporative cooling systems could influence their performance.

As a general conclusion, we recommend minimizing the flow rate to enhance the evaporative cooling system’s performance. Moreover, although such performance was less favorable when using DW foam material than when using DB foam material, in both cases, the requirements for the production of evaporative cooling systems were met. Consequently, these results highlight the feasibility of using polymer foam via additive manufacturing technology for the fabrication of efficient evaporative cooling systems.

Finally, as future work, based on the results of this research, we propose manufacturing a prototype of an evaporative cooling system using the optimal material identified in this research and analyzing its performance experimentally. This approach will allow the determination of the impact of the material on the energy performance, cost, and environmental impact of the system.

## Figures and Tables

**Table 1:** Design of experiments of process parameters for both foam materials.

Sample	D_ref_	D1	D2	D3	D4	D5	D6	D7	D8	D9
Sample reference	DW_ref_ DB_ref_	DW1 DB1	DW2 DB2	DW3 DB3	DW4 DB4	DW5 DB5	DW6 DB6	DW7 DB7	DW8 DB8	DW9 DB9
LW (mm)	0.4	0.4	0.4	0.4	0.6	0.6	0.6	0.8	0.8	0.8
F (%)	100	50	70	90	50	70	90	50	70	90
S (mm/s)	40	40	60	80	60	80	40	80	60	40
LH (mm)	0.2	0.2	0.3	0.4	0.4	0.2	0.3	0.3	0.4	0.2
T (°C)	210	250	250	250	250	250	250	250	250	250

**Table 2:** *p*-values obtained from the statistical analysis of the DOE results.

	CH-DW	I_m_-DW	CH-DB	I_m_-DB
LW (mm)	0.006	0.033	0.582	0.542
F (%)	0.005	0.003	0.047	0.008
S (mm/s)	0.378	0.375	0.567	0.729
LH (mm)	0.017	0.154	0.963	0.736

**Table 3:** Pore radius estimated from capillary rise values.

Specimen	CH [mm]	$R_{pore}$$\;\mathrm{Estimated}\;\mathrm{from}\;\mathrm{Jurin}’s\;\mathrm{Law}\;(\mathrm{Assuming}\;\theta_{Y}$ = 60°) [μm]	Macropores at Visual Observation?
DW1	85	86	yes
DW2	86	85	yes
DW3	87	84	yes
DW4	45	163	yes
DW5	80	92	yes
DW6	100	73	yes
DW7	47	156	yes
DW8	44	167	yes
DW9	83	88	yes
DW_ref_	20	367	no
DB1	69	106	yes
DB2	15	489	no
DB3	8	917	no
DB4	53	138	yes
DB5	45	163	yes
DB6	16	459	no
DB7	41	179	yes
DB8	59	124	yes
DB9	18	407	no
DB_ref_	11	667	no

**Table 4:** Results of N_2_ adsorption isotherm tests for both foam materials.

Parameter	DW1	DW6	DW7	DB1	DB6	DB7
SA (m^2^/g)	1.17	0.54	0.86	0.81	0.42	0.80
PVA (cm^3^/g)	0.00087	0.00041	0.00071	0.00061	0.00032	0.00057
PVD × 10^5^ (cm^3^/g)	22	12	15	14	6	13
APWA (Å)	29.73	30.05	32.71	31.54	30.79	28.16
APWD (Å)	7.55	8.41	6.75	6.93	5.74	6.45

**Table 5:** Wet fraction values estimated from apparent contact angles according to the Cassie–Baxter model.

Specimen	*θ_CB_* [°]	f [-]
DW1	105.2	0.492
DW6	83.2	0.746
DW7	111.1	0.427
DB1	100.8	0.542
DB6	78.8	0.796
DB7	108.2	0.458

## Data Availability

The original contributions presented in this study are included in this article; further inquiries can be directed to the corresponding author.
